# A summary index of infant and child feeding practices is associated with child growth in urban Shanghai

**DOI:** 10.1186/1471-2458-12-568

**Published:** 2012-07-28

**Authors:** Jing-Qiu Ma, Li-Li Zhou, Yan-Qi Hu, Jin-Rong Liu, Shan-Shan Liu, Jie Zhang, Xiao-Yang Sheng

**Affiliations:** 1 Department of Child and Adolescent Healthcare, Xinhua Hospital Affiliated to Shanghai Jiaotong University School of Medicine, Shanghai Key Laboratory of Pediatric Gastroenterology and Nutrition, MOE-Shanghai Key Laboratory of Children’s Environmental Health, Shanghai, 200092, China

**Keywords:** Child feeding practice, Brief recalls, Anthropometric indices, Repeatability coefficient

## Abstract

**Background:**

Recently, an infant and child feeding index (ICFI) constructed on brief recalls of breastfeeding, feeding frequency and food diversification was assumed to provide long-term prediction about child feeding practices. The aim of this study was to investigate the association between the cross-sectional ICFI (CS-ICFI) or longitudinal ICFI (L-ICFI) and child anthropometric indices in downtown Shanghai, China.

**Methods:**

The prospective cohort study included 180 infants aged 5-7 mo with their main caregivers who were visited 3 times every 6 months over 12 months. A CS-ICFI was constructed for each visit by using data on feeding practices based on 24-h and 7-d recalls. An L-ICFI was constructed with use of the 3 CS-ICFIs. The associations between ICFI and length-for-age z score (LAZ), weight-for-age z score (WAZ), and weight-for-length z score (WLZ) were examined. The stability of the CS-ICFI was assessed by using repeatability coefficient (RC).

**Results:**

The L-ICFI was positively associated with LAZ and WAZ at Visit 3(*beta* = 0.151, *P* = 0.040 and *beta* = 0.173, *P* = 0.024, respectively). Moreover, the CS-ICFI at Visit 1 was positively associated with LAZ, WAZ and WLZ (*beta* = 0.160, *P* = 0.029; *beta* = 0.191, *P* = 0.009; *beta* = 0.176, *P* = 0.020) at Visit 3, and the CS-ICFI at Visit 3 was also positively associated with LAZ (*beta* = 0.176, *P* = 0.016). Stability of the CS-ICFI was shown by the value of 0.14 (95% CI: 0.07, 0.31) of the RC, which differed significantly from 0 (P < 0.05).

**Conclusions:**

The ICFI constructed on brief recalls based on cross-sectional studies can be used to evaluate the effects of child feeding practice on child growth.

## Background

Infant and young child feeding is of great importance for their nutrition and health. Problematic feeding practices such as inappropriate duration of breastfeeding and introduction of complementary feeding can not only lead to undernutrition, but also initiate the problem of overweight and obesity [1]. Consequently, effective feeding index is necessary in measuring the caregiver’s feeding behaviors and the infant and young child’s diet quality and quantity.

Recently, infant and child feeding practices have received increasing attention and further been assessed with the use of a summary index as shown in several studies [2-4]. This cross-sectional infant and child feeding index (CS-ICFI) included information on current breastfeeding and bottle feeding, feeding frequency (in the past 24 h), and 24-h or 7-d recalls of food diversification. The method of combining various aspects of infant and young child feeding into an age-specific CS-ICFI provides an optimal solution for variation of recommended practices within age ranges. In addition, because the feeding practices used by caregivers at the time of study were likely to have been followed for a long time, it was assumed that the ICFI constructed by using data collected with a brief recall can provide information about more lasting processes and thus, can be used to evaluate its relationship with linear growth.

Recent studies focused primarily on analyzing the association of CS-ICFI with infant growth measured by length-for-age z score (LAZ). However, results were inconsistent, with some studies finding a positive association [5-7], whereas others did not [8]. Furthermore, some researchers [9] built a longitudinal ICFI (L-ICFI) with summary of the information from several CS-ICFIs, and found it can provide information about feeding in a long term and associated with LAZ.

To our knowledge, the ICFI has so far been used mostly in the context of African and Latin American countries. Hence, it is important to validate the index in other regions with different socio-economic and cultural context. A previous cross-sectional survey of ICFI has been carried out in China’s rural areas and found the association between ICFI and WAZ and WLZ [6], however, this is the first study among infants and young children in an affluent metropolitan city.

The aim of our study is to assess the applicability of CS-ICFI to Chinese diets and to available data in a very different Asian context, and to further construct L-ICFI which summarizes the information from several repeated CS-ICFIs. At last, we evaluate the association between cross-sectional or longitudinal index and infant and child growth.

## Methods

### Study design and participants

This study was conducted in downtown area of Shanghai, Kongjiang community, which is a typical community in Shanghai. Kongjiang Community Health Center routinely provides basic medical services to the local community, including well-child checks for the community’s infants and young children.

From November 2008 to April 2010, all 6-month-old infants and their parents or main caregivers entering the Kongjiang Community Health Center for a routine well-child check were invited to take part in this study.

Eligibility criteria for infants included healthy singleton infants, 6-month-old (5- to 7- month-old), born between 37 and 42 weeks gestational age, with birth weight between 2,500 to 4,000 g, with no metabolic or physical problems.

295 infants aged 5-7 mo met the eligibility criteria. Eighteen mothers refused to participate and a total of 277 infants and their parents or main caregivers were included in the study at baseline and followed every 6 months, revisited around 12-month-old (10- to14-month-old) and 18-month-old (16- to 20-month-old). Questions about the main caregiver’s education, mother’s pre-pregnancy weight and height, family income were included in the baseline questionnaire. Infants feeding practices and consumption of food groups during the week preceding each visit were also assessed with questionnaires. Anthropometrics at each visit were measured. There were 29 infants had incomplete data at Visit 2, of whom the main caregivers of 2 infants refused to complete the questionnaire due to a lack of time and 27 infants had missing information in the questionnaire. There were 68 infants had incomplete data at Visit 3, of whom the main caregivers of 21 infants refused to complete the questionnaire because of a lack of time or impatience and 47 infants had missing information in the questionnaire. Only infant with a complete questionnaire and anthropometric data set of 3 visits were included in analysis, leaving a total of 180 infants. Infants were enrolled after parents or main caregivers provided informed consent. The study protocol was approved by the Ethics Committee of Xinhua Hospital, affiliated to Shanghai Jiao Tong University School of Medicine.

### Data collection

The weight and length of infants at each visit were measured using standardized procedures in community health center. Weight was measured using an electronic pediatric scale to the nearest 0.01 kg, and length was measured using a pediatric-length board with the infant in a recumbent position. LAZ, weight-for-age z scores (WAZ), weight-for-length z scores (WLZ) were calculated according to the 2006 WHO Child Growth Standards using the WHO Anthro 2009 software [10].

Underweight, stunting and wasting were defined as WAZ < -2, LAZ < -2, and WLZ < -2, respectively. Overweight was defined as WLZ > +2.

The information of infant feeding practices was obtained based on 24-h and 7-d food recalls through on-site face-to-face interviews with the parents or main caregivers by a trained clinical research assistant at each visit.

### Infants and child feeding index (ICFI)

The cross sectional infants and child feeding index (CS-ICFI) was constructed on the basis of current feeding practice for infants and young children recommended by WHO [11,12], as well as the methods proposed by other studies [2-4] taking into account local characteristics.

In our study, the CS-ICFI was defined for 3 different age groups: 6-month (5- to 7-month), 12-month (10- to 14-month), and 18-month (16- to 20-month), respectively. The following 5 variables were used in the index construction: breastfeeding, food consistency, feeding frequency, dietary diversity score (DDS), and food group frequency score (FGFS), which were attributed in a manner that reflected the age change over the study period.

Table 1 lists variables and the scoring system used to create the CS-ICFI for the different age groups. In particular,

**Table 1:** Variables and scoring system used to construct the infant and child feeding index (CS-ICFI)

	**scores**		
**Variables**	**Visit 1 (6-month-old)**	**Visit 2 (12-month-old)**	**Visit 3 (18-month-old)**
Breastfeeding	Yes = +2	Yes = +1	Yes = 0
	No = 0	No = 0	No = 0
Food consistency ^a^	Liquid = 0	Gruel-like = 0	Gruel-like = 0
	Gruel-like = +1	Semi-solid = +1	Semi-solid = +1
	Semi-solid = +2	Solid = +2	Solid = +2
Feeding frequency (past 24-h) ^b^	No = 0	0-2 times =0	0-2 times =0
	1 times = +1	3-4 times or ≧9 times = +1	3-4 times = +1
	≧2 times = +2	5-8 times = +2	≧9 times = +2
			5-8 times = +3
Food group frequency score (FGFS) (past 7-d)	FGFS 0 ~ 3 = 0	FGFS 0 ~ 5 = 0	FGFS 0 ~ 7 = 0
	FGFS 4 ~ 9 = +1	FGFS 6 ~ 9 = +1	FGFS 8 ~ 11 = +1
	FGFS 10 ~ 16 = +2	FGFS 10 ~ 12 = +2	FGFS 12 ~ 14 = +2
		FGFS 13 ~ 16 = +3	FGFS 15 ~ 16 = +3
Dietary diversity score(DDS) (past 7-d)	DDS 0 ~ 2 = 0	DDS 0 ~ 3 = 0	DDS 0 ~ 4 = 0
	DDS 3 ~ 4 = +1	DDS 4 ~ 6 = +1	DDS 5 ~ 7 = +1
	DDS 5 ~ 8 = +2	DDS 7 ~ 8 = +2	DDS 8 = +2
Total score	10 points	10 points	10 points

^a^Food consistency was the highest consistency of complementary food.

^b^For Visit 1, feeding frequency of breastfeeding and formula-feeding was excluded; for Visits 2 and 3, the times of taking all food groups consumed above 10 g were counted.

#### Breastfeeding

A score of +2, +1 was given to 6-, 12-month-old infants who were breastfed respectively. Among young children aged 18 months, only 3 were breastfed, and for elder child, complementary feeding is considered more important than breastfeeding, so a score of 0 was given to 18-month-old young children who were breastfed. A score of 0 was given to non-breastfeeding infants at all ages.

#### Food consistency

Food consistency was used as a component of CS-ICFI instead of bottle feeding because bottle use is considered potentially harmful for all children throughout the age range included. Food consistency was defined as the highest consistency of complementary food. For 6-month-old infants, liquid food was given a score of 0, gruel-like food +1, and semi-solid food +2. For 12- and 18-month-old infants, gruel-like food, semi-solid food, and solid food were scored as 0, +1, and +2, respectively.

#### Feeding frequency

Feeding frequency means how many times the infants was offered different foods in the previous 24 h. For 6-month-old infants, feeding frequency was used to account the times for complementary foods excluding breastfeeding and formula-feeding. None (meaning no complementary foods) was given a score of 0, 1 time a day received a score of +1 and 2 times or more +2. For 12- and 18-month-old infants, the times of taking all food groups consumed above 10 g were counted as shown in Table 1.

#### Dietary diversity score (DDS)

DDS was calculated on the basis of the number of different food groups infants consumed over the past 7 days. Eight food groups were taken into account which included the major nutritionally important types of food the infants may have eaten in our context, as follows: 1) starchy staples (foods made from grain, roots, or tubers); 2) legumes and nuts; 3) dairy (milk other than breast milk, cheese, or yogurt); 4) meat, poultry, or liver/organ meats 5) fish, shrimp or crabs; 6) eggs; 7) fruits and 8) vegetables. Each food group consumed by infants during the previous week was scored as 1. These scores were summed to give a possible range of 0-8, and then new scores were assigned as described in Table 1 according to different age distribution.

#### Food group frequency score (FGFS)

FGFS was assessed based on the frequency of different food groups consumed by infants over the past 7 days. FGFS was scored as 0 if not consumed during the previous week, +1 if consumed on 1-3 days, and +2 if consumed on ≧4 days. These scores were summed to give a possible range of 0-16, and then new scores were assigned as described in Table 1 to reflect age-specific distribution.

The final CS-ICFI was a summation of the scores obtained for each variable described above. The index ranged from 0 to 10 for all three age groups.

The CS-ICFI was divided into 3 categories based as closely as possible on tertiles with all age groups together in the following manner: a score of 0-7 was considered low, 8 was considered medium, and 9-10 was considered high.

Another index was constructed to summarize the information from the 3 CS-ICFIs corresponding to the 3 visits. Each CS-ICFI was coded 0 for low, 1 for medium, and 2 for high. These scores were summed over the 3 visits for each infant to give a possible range of 0-6. The so-called continuous L-ICFI was then cut based on tertiles in the following manner: 0-2 was considered low, 3 was considered medium, and 4-6 was considered high [9].

### Statistical analysis

The associations between the CS-ICFIs or L-ICFI with LAZ, WAZ and WLZ were analysed using anthropometric data from visit 3. Linear regression models was used to analyze the association of each component of the CS-ICFIs with LAZ, WAZ and WLZ at Visit 3, and the association of L-ICFI with LAZ, WAZ and WLZ at Visit 3. For each model, birth weight, mother’s pre-pregnancy height and weight, and annual household income were adjusted as confounders. The association between CS-ICFIs and LAZ, WAZ and WLZ at Visit 3 was also examined by a linear regression model, and child sex and age, birth weight, mother’s pre-pregnancy height and annual household income were adjusted as confounders.

Changes in the distribution of CS-ICFI components over time were examined at the group level. Chi-square test was used to explore whether the distribution of the CS-ICFI components differed between different visits.

To examine the longitudinal changes of CS-ICFI and eventually obtain L-ICFI value for each infant we had to take into account a disturbing factor: that the CS-ICFI scoring system does change across age categories. Therefore, first, to make CS-ICFI values comparable over time we decided to normalize their distribution and to use the z scores instead of original CS-ICFI values. Second, we were challenged by the choice of a statistical model to test whether these CS-ICFI z values were stable for each infant from one visit to the other. To estimate the stability of 3 visits, we used repeatability coefficient (RC) which was also referred to as interclass correlation coefficient proposed by Moursi [9]. RC [13] is the ratio of the between-subject variance divided by the sum of the between-subject and the within-subject variance applied to test the repeatability of measures from the same subject. The variance for the 3 visits’ CS-ICFI z values of the same infant was considered conceptually an equivalent of the within-subject part of the total variance of the measurement which is recommended to estimate the measurement error [14]. Because we used normalized values (z scores), the total variance of all the CS-ICFI z values was 1. RC ranges between 0 and 1, with RC = 0 indicating no repeatability at all (absence of stability from visit to visit) and RC = 1 indicating perfect repeatability (perfect stability from visit to visit). Thus, it is possible to test whether the RC is significantly different from zero.

All analyses were performed by using SPSS 13.0 for windows and statistical significance was set at 0.05.

## Results

### Loss to follow-up

There were no significant differences in mean LAZ (*P* = 0.217), mean WAZ (*P* = 0.346), mean WLZ (*P* = 0.689), mean age (*P* = 0.062), and sex ratio (*P* = 0.457) of the 29 infants whose caregivers did not complete the questionnaires versus the infants with complete data set at Visit 2. Similarly, the 68 infants who had uncompleted questionnaires at Visit 3 did not differ significantly from the remaining sample with respect to mean LAZ (*P* = 0.779), mean WAZ (*P* = 0.510), mean WLZ (*P* = 0.453), mean age (*P* = 0.217), and sex ratio (*P* = 0.091) at Visit 3.

### Sample characteristics

Main sample characteristics of 3 visits are shown in Table 2. The mean ages of these 180 infants at 3 visits were (5.85 ± 0.44), (12.13 ± 0.29), and (18.12 ± 0.27) months, respectively. Among them, there were 79 boys (43.89%) and 101 girls (56.11%). Most children grew well in this community. Evaluated by the WHO Child Growth Standards, child mean LAZ, WAZ and WLZ of 3 visits were all above the WHO median. There were only one infant (0.38%) stunting (LAZ < -2) at Visits 2 and 3, and another one (0.38%) wasting (WLZ < -2) at Visit 3. There was no underweight (WAZ < -2) infants over the study period. However, the rates of overweight (WLZ > +2) of infants at 3 visits were 6.11%, 5.00% and 5.00%, respectively.

**Table 2:** Main characteristics of the cohort

**Characteristic**	**n**	**result**
Child age		
Visit 1 (6-month-old)	180	5.85 ± 0.44 ^a^
Visit 2 (12-month-old)	180	12.13 ± 0.29
Visit 3 (18-month-old)	180	18.12 ± 0.27
Child gender [n (%)]		
Male	79	43.89
Female	101	56.11
Breastfeeding [n (%)]		
Visit 1 (6-month-old)	88	48.89
Visit 2 (12-month-old)	12	6.67
Visit 3 (18-month-old)	3	1.67
Child nutritional status		
Length-for-age z score		
Visit 1 (6-month-old)	180	0.58 ± 0.80
Visit 2 (12-month-old)	180	0.43 ± 0.88
Visit 3 (18-month-old)	180	0.41 ± 0.91
Weight-for-age z score		
Visit 1 (6-month-old)	180	0.83 ± 0.78
Visit 2 (12-month-old)	180	0.70 ± 0.74
Visit 3 (18-month-old)	180	0.65 ± 0.80
Weight-for-length z score		
Visit 1 (6-month-old)	180	0.76 ± 0.88
Visit 2 (12-month-old)	180	0.70 ± 0.77
Visit 3 (18-month-old)	180	0.62 ± 0.82
Mother’s pre-pregnancy height (cm)	180	161.61 ± 4.24
Mother’s pre-pregnancy weight (kg)	180	55.64 ± 8.02
Mother’s pre-pregnancy BMI (kg/m^2^)	180	21.30 ± 2.93
Caregiver’s schooling years [n (%)]		
≦12y	75	41.67
>12y	105	58.33
Annual household income [n (%)]		
≦RMB 60 thousand per year	56	31.11
RMB 60-150 thousand per year	81	45.00
>RMB 150 thousand per year	43	23.89

^a^Mean ± SD (all such values).

Breastfeeding was relatively common during the first 6 months but did not last into the second year of life; 48.89% of infants in our sample were breastfed at baseline, but at Visit 3, only 1.67% was breastfed.

### Distribution of the CS-ICFI components at different time points

Many of the components of the CS-ICFI varied with time at the group level (Table 3). As expected, breastfeeding decreased over time as infants grew. At the same time, food consistency changed with infants receiving more semi-solid and solid food. The percentage of infants in the high feeding frequency category moved from 89.44% to 100% between Visit 1 and Visit 3. Dietary diversity and food group frequency also changed in a positive direction. The percentage of infants with high category rose from 63.33% to 90.00% and from 46.11% to 98.33%, respectively, over the study period.

**Table 3:** Distribution of the CS-ICFI components at different time points

**Components**	**score**	**Visit 1 [n(%)]**	**Visit 2 [n(%)]**	**Visit 3 [n(%)]**	***P*value**^**a**^
Breastfeeding					
Yes		88 (48.89)	12 (6.67)	3 (1.67)	<0.0001
Food consistency					
Low	0/+1	105 (58.33)	79 (43.89)	10 (5.56)	<0.0001
High	+2	75 (41.67)	101 (56.11)	170 (94.44)	
Feeding frequency					
Low	0/+1	19 (10.56)	54 (30.00)	0 (0.00)	<0.0001
High	+2/+3	161 (89.44)	126 (70.00)	180 (100.00)	
Food group frequency					
Low	0/+1	97 (53.89)	1 (0.56)	3 (1.67)	<0.0001
High	+2/+3	83 (46.11)	179 (99.44)	177 (98.33)	
Dietary diversity					
Low	0/+1	66 (36.67)	9 (5.00)	18 (10.00)	<0.0001
High	+2	144 (63.33)	171 (95.00)	162 (90.00)	

^a^Chi-square test.

### Association between CS-ICFIs and LAZ, WAZ and WLZ at Visit 3

The CS-ICFI at Visit 1 (6-month-old) was positively associated with child LAZ, WAZ and WLZ (*beta* = 0.160, *P* = 0.029; *beta* = 0.191, *P* = 0.009 and *beta* = 0.176, *P* = 0.020, respectively) at Visit 3(18-month-old). Furthermore, the CS-ICFI at Visit 3 was also positively associated with child LAZ (*beta* = 0.176, *P* = 0.016) at Visit 3. However, there was no significant association between the CS-ICFI at Visit 2(12-month-old) and LAZ, WAZ and WLZ at Visit 3 after adjustment for confounders by a linear regression model (Table 4).

**Table 4:** Association between CS-ICFIs and LAZ, WAZ and WLZ at Visit 3

**CS-ICFIs**		**LAZ**	**WAZ**	**WLZ**
Visit 1 (6-month-old)	*Beta*^a^	0.160	0.191	0.176
	*Std.Error*	0.042	0.037	0.040
	*P* value ^b^	0.029	0.009	0.020
Visit 2 (12-month-old)	*Beta*^a^	-0.071	0.011	0.065
	*Std.Error*	0.079	0.072	0.074
	*P* value ^b^	0.326	0.884	0.387
Visit 3 (18-month-old)	*Beta*^a^	0.176	0.042	-0.066
	*Std.Error*	0.073	0.070	0.074
	*P* value ^b^	0.016	0.585	0.398

^a^Standardized partial regression coefficient.

^b^Association between CS-ICFI and LAZ, WAZ and WLZ at each time point. adjusted for child sex and age, birth weight, mother’s pre-pregnancy height and annual household income using a linear regression model.

### Association between L-ICFI and LAZ, WAZ and WLZ at Visit 3

Mean LAZ and WAZ at Visit 3 (18-month-old) increased as scores of the L-ICFI increased and the association was positively significant after adjustment for confounders (*beta* = 0.151, *P* = 0.040 and *beta* = 0.173, *P* = 0.024, respectively). There was a difference of 0.26 in mean LAZ when moving from the low to the high category of the L-ICFI. However, the mean WLZ had no relationship with the L-ICFI values (Table 5).

**Table 5:** Association between L-ICFI and LAZ, WAZ and WLZ at Visit 3 (18-month-old)

**Variables**	**Score (0 ~ 6)**	**n**	**Mean LAZ**^**a**^	**Mean WAZ**^**a**^	**Mean WLZ**^**a**^
L-ICFI					
Low	0 ~ 2	43	0.23	0.62	0.69
Medium	3	57	0.35	0.47	0.41
High	4 ~ 6	80	0.49	0.79	0.76
*Beta*^b^			0.151	0.173	0.121
*Std.Error*			0.051	0.047	0.050
*P* value ^c^			0.040	0.024	0.119

^a^Adjusted for mother’s pre-pregnancy height and weight, birth weight and annual household income.

^b^Standardized partial regression coefficient.

^c^Associations between L-ICFI and mean LAZ, WAZ and WLZ were tested using a linear regression model.

### Association of the CS-ICFIs components at each time point with LAZ, WAZ and WLZ at Visit 3

Among the components of the CS-ICFI at Visit 1 (6-month-old), food consistency was the only component significantly associated with child LAZ (*beta* = 0.171, *P* = 0.021) and WAZ (*beta* = 0.192, *P* = 0.013) at Visit 3(18-month-old) (Table 6).

**Table 6:** Association between the components of CS-ICFI at Visit 1 (6-month-old) and LAZ, WAZ, WLZ at Visit 3 (18-month-old)

**Variables**	**Score**	**n**	**Mean LAZ**^**a**^	**Mean WAZ**^**a**^	**Mean WLZ**^**a**^
Breastfeeding					
No	0	92	0.33	0.58	0.58
Yes	+2	88	0.44	0.72	0.69
*Beta*^c^			0.063	0.087	0.070
*Std.Error*			0.066	0.061	0.064
*P* value ^d^			0.391	0.255	0.364
Food consistency					
Low	0	17	0.20	0.35	0.35
Median	+1	88	0.26	0.57	0.60
High	+2	75	0.58	0.81	0.73
*Beta*^c^			0.171	0.192	0.130
*Std.Error*			0.102	0.094	0.100
*P* value ^d^			0.021	0.013	0.097
Feeding frequency ^b^					
Low	0/+1	19	0.36	0.49	0.45
High	+2	161	0.39	0.67	0.66
*Beta*^c^			0.012	0.070	0.079
*Std.Error*			0.217	0.201	0.211
*P* value ^d^			0.874	0.376	0.323
Dietary diversity					
Low	0	11	0.44	0.58	0.51
Median	+1	55	0.20	0.52	0.57
High	+2	114	0.46	0.71	0.67
*Beta*^c^			0.087	0.096	0.063
*Std.Error*			0.111	0.103	0.109
*P* value ^d^			0.245	0.216	0.422
Food group frequency ^b^					
Low	0/+1	97	0.36	0.63	0.62
High	+2	83	0.41	0.67	0.64
*Beta*^c^			0.039	0.051	0.037
*Std.Error*			0.119	0.111	0.116
*P* value ^d^			0.604	0.515	0.641

^a^Adjusted for birth weight, mother’s pre-pregnancy height and weight, and annual household income.

^b^Samples with scores of 0 and +1 were combined due to the extremely small size of sample with score 0.

^c^Standardized partial regression coefficient.

^d^Associations between the components of CS-ICFI at Visit 1 and mean LAZ, WAZ and WLZ at Visit 3 were tested using a linear regression model.

Among the components of the CS-ICFI at Visit 3, both dietary diversity and food group frequency score were significantly associated with child LAZ (*beta* = 0.156, *P* = 0.036 and *beta* = 0.171, *P* = 0.021, respectively) at Visit 3. There were 0.47 and 0.29 mean LAZ differences from low to high score category of dietary diversity and food group frequency, respectively. Food consistency was significantly associated with WAZ (*beta* = 0.167, *P* = 0.029) and WLZ (*beta* = 0.163, *P* = 0.034). Infants in high score categories had higher mean WAZ and WLZ, and the differences were 0.57 and 0.58, respectively (Table 7).

**Table 7:** Association between the components of CS-ICFI at Visit 3 and LAZ, WAZ, WLZ at Visit 3 (18-month-old)

**Variables**	**Score**	**n**	**Mean LAZ**^**a**^	**Mean WAZ**^**a**^	**Mean WLZ**^**a**^
Food consistency					
Low	+1	10	0.10	0.11	0.09
High	+2	170	0.40	0.68	0.67
*Beta*^c^			0.079	0.167	0.163
*Std.Error*			0.280	0.257	0.270
*P* value ^d^			0.280	0.029	0.034
Feeding frequency ^b^					
Low	+1/+2	51	0.39	0.71	0.71
High	+3	129	0.39	0.63	0.60
*Beta*^c^			-0.002	-0.047	-0.059
*Std.Error*			0.009	0.137	0.144
*P* value ^d^			0.982	0.541	0.452
Dietary diversity					
Low	+1	18	-0.04	0.44	0.62
High	+2	162	0.43	0.67	0.63
*Beta*^c^			0.156	0.085	0.004
*Std.Error*			0.221	0.207	0.218
*P* value ^d^			0.036	0.274	0.959
Food group frequency ^b^					
Low	+1/+2	97	0.25	0.69	0.78
High	+3	83	0.54	0.60	0.47
*Beta*^c^			0.171	-0.034	-0.164
*Std.Error*			0.123	0.116	0.121
*P* value ^d^			0.021	0.658	0.035

^a^Adjusted for birth weight, mother’s pre-pregnancy height and weight, and annual household income.

^b^Samples with scores of +1 and +2 were combined due to the extremely small size of sample with score +1.

^c^Standardized partial regression coefficient.

^d^Associations between the components of CS-ICFI at Visit 3 and mean LAZ, WAZ and WLZ at Visit 3 were tested using a linear regression model.

However, there was no significant association between the components of the CS-ICFI at Visit 2 (12-month-old) and child anthropometric indices at Visit 3.

### CS-ICFI distribution at different visits

The mean and distribution of the original CS-ICFI values according to different visits are shown in Table 8. The change of age and CS-ICFI scoring system which influenced the original CS-ICFI values resulted in the variation in subjects and methods. The approach with the use of CS-ICFI z scores instead of original CS-ICFI values led to an estimate of the repeatability coefficient (RC) of 0.14 (95% CI: 0.07, 0.31), which was significantly different from zero (*P* < 0.05). It indicated that the CS-ICFI z values were stable for each child from one visit to the other.

**Table 8:** CS-ICFI distribution by visit (n = 180)

**CS-ICFI**	**Visit 1 (6-month-old)**	**Visit 2 (12-month-old)**	**Visit 3 (18-month-old)**
Mean	7.19	7.99	9.01
SD	1.55	0.83	0.87
Median	7	8	9
Minimum	3	5	6
maximum	10	10	10

## Discussion

### Association of ICFI with Child Growth Status

This study constructed modified CS-ICFIs taking into consideration of Chinese diet characteristics for 6- to 18-month-old infants by 3 different age groups, meanwhile attempted to create an L-ICFI by using data from CS-ICFIs, and further analyzed the association between CS-ICFIs or L-ICFI and infants’ growth status.

Despite the homogeneous urban context, our results showed that the CS-ICFI of 18-month-old children at Visit 3 was significantly and positively associated with their LAZ at the same visit. Our findings confirmed the conclusion that ICFI was significantly associated with LAZ especially among 12- to 36-month-old children based on the data from 7 Demographic and Health Surveys in 5 Latin American countries [2]. Furthermore, we also found that the CS-ICFI of 6-month-old infants at Visit 1 was significantly and positively associated with their LAZ, WAZ and WLZ measured again a year later at Visit 3. It is suggested that previous feeding practice may have long-term effect on future growth of children. However, there was no significant association between the CS-ICFI of 12-month-old infants and anthropometric indices measured 6 months later at Visit 3.

Next, our analysis indicated that longitudinal ICFI constructed using data from the 3 CS-ICFIs was positively and significantly associated with LAZ. It corresponded with the result reported by a recent study [9]. In addition, we also found the significant association between L-ICFI and WAZ similar to the result reported in a previous study performed in rural China [6]. Infant and young child feeding practices varying with regions, ethnicities, diet cultures and socio-economy due to various study settings might contribute to the difference in results.

### Components of CS-ICFI

The percentage of breastfeeding decreased drastically from 48.89% for 6-month-old infants to 1.67% for 18-month-old infants. Only 3 young children were breastfed with the frequency of < 3 times per day at Visit 3 and thus breastfeeding had very little effect on the growth of these children. As a result, we had to remove breastfeeding from the components of CS-ICFI at Visit 3.

Secondly, bottle feeding will interfere with optimal breastfeeding practices, reducing breast milk intake and energy intake from complementary food, as well as increasing risk of infection [6,15]. On the other side, food consistency can indirectly reflect the frequency of bottle feeding because bottle use is limited by the texture of food. Furthermore, food consistency can also reflect the energy density of complementary food. Taking these factors into account, bottle feeding was replaced by food consistency in our study. Our results showed that consistency of complementary food of ICFI at Visit 1 was positively associated with child LAZ and WAZ at Visit 3, and at Visit 3, 18-month-old infants in high score categories of food consistency had over 0.55 higher mean WAZ and WLZ, respectively. Though food consistency taking the place of bottle feeding is an innovative attempt, however, it raises some questions. First, the majority of young children aged 18 months receiving solid staple food resulted in small sample size in the low food consistency group which might affect the accuracy of the statistical results. Second, high food consistency generally accompanied with high energy density may offer more energy intakes [16]. Food consistency used as a component of CS-ICFI may drive the relationship of L-ICFI with WAZ. Therefore, food consistency score should be used with caution and needs to be investigated in future studies.

It has been pointed out that a 24-h recall cannot appropriately reveal the actual quality of the diet, mainly because of possible variations from one day to another [3]. In our context, due to some special Chinese diet habits, food groups varied not much within 24 h, so it was better to count number of food groups consumed based on the information collected from the past 7 days. Therefore, we tested a slightly modified version of dietary diversity based on 7-d recalls, which actually has been applied in two recent studies [8,17]. Several studies have illustrated that food diversity was a good indicator of adequate micronutrient intake and associated with child nutritional status [18-20]. Our findings also confirmed that both dietary diversity and food group frequency score were associated with LAZ among 18-month-old infants. Furthermore, it is suggested that both dietary diversity and food group frequency score are likely driving the relationship of L-ICFI with LAZ.

### Challenges in constructing a longitudinal ICFI

The first consideration in constructing a longitudinal ICFI is the stability of CS-ICFIs over the study period at the individual level.

To test the stability, we applied repeatability coefficient proposed by Moursi [9], which was also referred to as interclass correlation coefficient and used to test the reproducibility of replicate measures from the same subject, with 0 indicating no stability over time at all and 1 indicating perfect stability from visit to visit. Fortunately, we found that the value of repeatability coefficient was 0.14 which differed significantly from zero. This value means that the CS-ICFI is relatively stable over time at least at the individual level and it is feasible to construct L-ICFI using the information from several CS-ICFIs.

Furthermore, changes in the distribution of the CS-ICFI components by different age groups were determinant for the relative stability of CS-ICFI. As expected, infants were breastfed less as they grew older, whereas feeding frequency, food consistency and food diversity all increased over time. In addition, due to the obvious decrease of breastfeeding as infants grew in our context, we had to modify the scoring methods of food group frequency and feeding frequency for elder infants which were not divided into tertiles to harmonize the whole scoring system of CS-ICFI. As a result, the decline of the CS-ICFI as breastfeeding dropped with age partly compensated for by higher feeding frequency, food consistency and food diversity led to the slight increase of CS-ICFI scores over time. Therefore, we acknowledged that the stability might not be very strong as Moursi reported in their study even if the RC was statistically significantly different from zero.

Despite the variations in the methods used to build CS-ICFI, and the limitation in the construction of L-ICFI, our results confirmed the existing literature and suggested that ICFI was positively associated with anthropometric indicators of nutritional status among infants and young children [2,3,9].

In addition, our study has a noteworthy finding. This is the first attempt to apply ICFI in an affluent downtown area in China different from the previous studies conducted in relatively impoverished settings. The rate of overweight of infants was higher in the surveyed area. The CS-ICFI at 6 months positively associated with not only child LAZ but also WAZ and WLZ at 18 months may be due to the affluent setting with high rate of overweight in infants, which was similar to the results in a study conducted in China’s rural areas[6]. However, there was no association between longitudinal ICFI and WLZ in our study suggested that ICFI was related with optimal nutritional status of infants and young children in the long term and might be used in more affluent areas in further studies.

Our study also has several limitations. First, the data were only collected from a community in Shanghai which could not represent the general conditions of child feeding practice in China. Secondly, because the caregivers reported about themselves with no objective reporters, the risk of reporting bias may exist. Additionally, there may be some unmeasured confounders which probably had some influence on the results.

## Conclusion

In conclusion, CS-ICFI at 6 months was positively associated with child growth status at 18 months indicated that previous feeding practice may predict future growth of children. Moreover, the longitudinal ICFI which was constructed with use of data collected from a brief recall based on cross-sectional studies was positively associated with LAZ and WAZ. It is suggested that ICFI can be used to evaluate the effects of child feeding practice on child growth for a longer time.

## Competing interests

The authors declare that they have no competing interests.

## Authors’ contributions

J-QM performed the acquisition of data, analysis of data and drafted the paper. L-LZ, Y-Q H and J-RL participated in the design of study, acquisition of data; S-SL and JZ contributed to the acquisition of data. X-YS was the chief of the study and responsible for the whole project, revising the paper and approving the final version to be submitted. All authors have read and have approved the manuscript as submitted.

## Pre-publication history

The pre-publication history for this paper can be accessed here:

http://www.biomedcentral.com/1471-2458/12/568/prepub
